# Supplementary material for: ZnO Nanostructures Doped with Various Chloride Ion Concentrations for Efficient Photocatalytic Degradation of Methylene Blue in Alkaline and Acidic Media
Source: Molecules. 2022 Dec 9;27(24):8726. doi: 10.3390/molecules27248726 (PMC9781204; doi:10.3390/molecules27248726)
Supplement: Supplementary file 1 [file molecules-27-08726-s001.zip › molecules-2047900-supplementary.pdf]

Supplementary Materials

ZnO Nanostructures Doped with Various Chloride Ion Concentrations for Efficient Photocatalytic Degradation of Methylene Blue in Alkaline and Acidic Media

Razan A. Alshgari [1], Zaheer Ahmed Ujjan [2,*], Aqeel Ahmed Shah [3], Muhammad Ali Bhatti [4], Aneela Tahira [5], Nek Muhammad Shaikh [2], Susheel Kumar [2], Mazhar Hussain Ibupoto [6], Amal Elhawary [7], Ayman Nafady [1], Brigitte Vigolo [8] and Zaffar Hussain Ibhupoto [9,*]

[1] Department of Chemistry, College of Science, King Saud University, Riyadh 11451, Saudi Arabia
[2] Institute of Physics, University of Sindh Jamshoro, Jamshoro 76080, Pakistan
[3] Department of Metallurgy, NED University of Engineering and Technology Karachi, Karachi 75270, Pakistan
4 Institute of Environmental Sciences, University of Sindh Jamshoro, Jamshoro 76080, Pakistan
[5] Institute of Chemistry, Shah Abdul Latif University Khairpur Mirs, Khairpur Mirs 66020, Pakistan
[6] Department of Zoology, Shah Abdul Latif University Khairpur Mirs, Khairpur Mirs 66020, Pakistan
[7] Department of Chemistry and physics, Faculty of Education, Alexandria University, Alexandria 21500, Egypt
[8] College of Natural Resources and Sciences, Jean Lamour Institute, Université de Lorraine, F-54000 Nancy, France
[9] Institute of Chemistry, University of Sindh Jamshoro, Jamshoro 76080, Pakistan
* Correspondence: zaheer.ujjan@scholars.usindh.edu.pk (Z.A.U.); zaffar.ibhupoto@usindh.edu.pk (Z.H.I.)

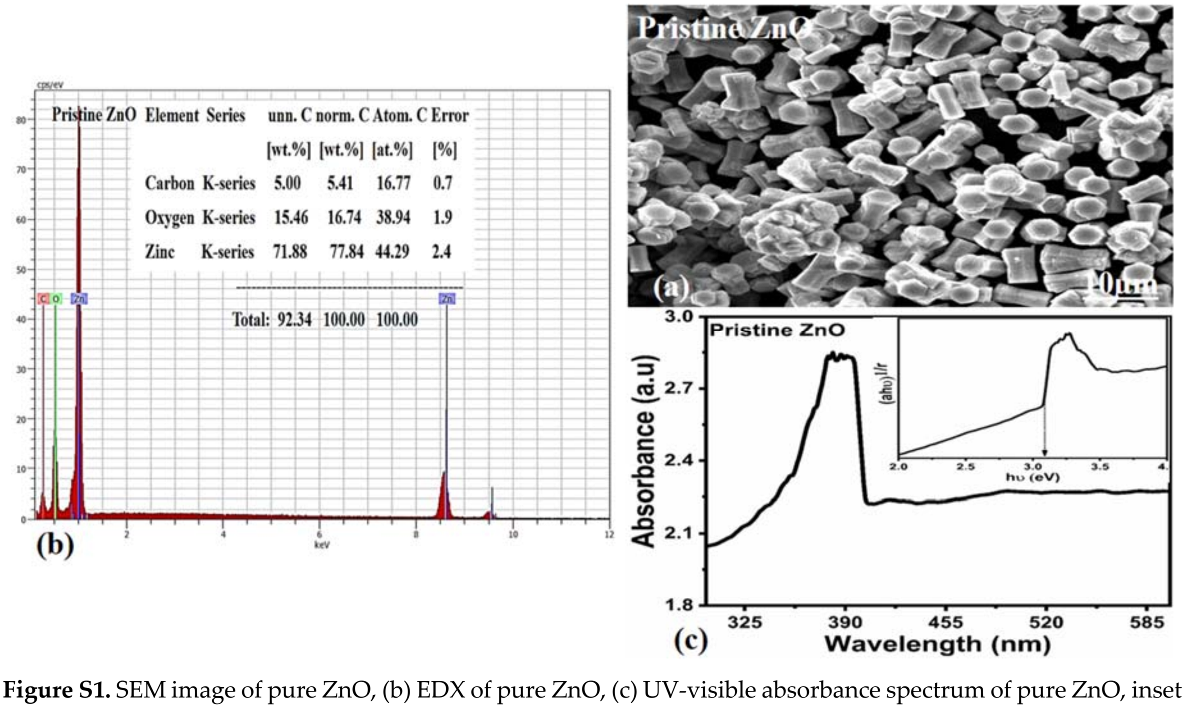

Figure S1. SEM image of pure ZnO, (b) EDX of pure ZnO, (c) UV-visible absorbance spectrum of pure ZnO, inset show the optical band gap.
